# Leukoplakia and Immunology: New Chemoprevention Landscapes?

**DOI:** 10.3390/ijms21186874

**Published:** 2020-09-19

**Authors:** Roberto Grigolato, Maria Eleonora Bizzoca, Luca Calabrese, Stefania Leuci, Michele Davide Mignogna, Lorenzo Lo Muzio

**Affiliations:** 1 Division of Prevention, San Maurizio Hospital, 39100 Bolzano, Italy; roberto.grigo@tiscali.it; 2 Department of Clinical and Experimental Medicine, University of Foggia, 71122 Foggia, Italy; marielebizzoca@gmail.com; 3 Division of Otorhinolaryngology, “San Maurizio” Hospital, 39100 Bolzano, Italy; dott.lucacalabrese@gmail.com; 4 Department of Neurosciences, Reproductive and Odontostomatological Sciences, Oral Medicine Unit, Federico II University of Naples, 80138 Naples, Italy; stefania.leuci@unina.it (S.L.); mignogna@unina.it (M.D.M.); 5 C.I.N.B.O. (Consorzio Interuniversitario Nazionale per la Bio-Oncologia), 66100 Chieti, Italy

**Keywords:** OPMDs, Leukoplakia, chemoprevention, immunology, cancer, HNSCC

## Abstract

Oral potentially malignant disorders (OPMDs) comprise a range of clinical-pathological alterations frequently characterized by an architectural and cytological derangements upon histological analysis. Among them, oral leukoplakia is the most common type of these disorders. This work aims to analyze the possible use of drugs such as immunochemopreventive agents for OPMDs. Chemoprevention is the use of synthetic or natural compounds for the reversal, suppression, or prevention of a premalignant lesion conversion to malignant form. Experimental and in vivo data offer us the promise of molecular prevention through immunomodulation; however, currently, there is no evidence for the efficacy of these drugs in the chemoprevention action. Alternative ways to deliver drugs, combined use of molecules with complementary antitumor activities, diet influence, and better definition of individual risk factors must also be considered to reduce toxicity, improve compliance to the protocol treatment and offer a better individualized prevention. In addition, we must carefully reconsider the mode of action of many traditional cancer chemoprevention agents on the immune system, such as enhancing immunosurveillance and reversing the immune evasion. Several studies emphasize the concept of green chemoprevention as an alternative approach to accent healthy lifestyle changes in order to decrease the incidence of HNSCC.

## 1. Introduction

Oral potentially malignant disorders (OPMDs) comprise a range of clinical-pathological alterations that are frequently characterized by an architectural and cytological derangements upon histological analysis. Among them, oral leukoplakia is the most common type of these disorders. Currently, the definition of *leukoplakia* is a “white plaque of questionable risk having excluded (other) known diseases or disorders that carry no increased risk for cancer” [1]. In addition, leukoplakia is primarily a clinical term and has no specific histology. 

According to a recent review by Warnakulasuriya et al., the estimated overall malignant transformation rate of leukoplakia is about 3.5% with a wide range between 0.13% and 34% [2]. Several factors have been involved in the etiology of leukoplakia, such as HPV. De La Cour et al. found an overall pooled HPV prevalence of 22.5% (95% confidence interval 16.6–29.0) across the review of 52 studies with 2677 cases [3].

Some authors noted a significant increment of CD8+ cells in OPMDs, such as leukoplakia, that evolved into carcinomas, suggesting a possible role of immunology in the transformation processes [4].

To date, there is insufficient evidence for prognostic biomarkers of oral leukoplakia [5]. Management and treatment of leukoplakia is still challenging, particularly for large lesions and the proliferative subtype [6]. Chemoprevention is the use of synthetic or natural compounds for the reversal, suppression, or prevention of a premalignant lesion conversion to an invasive form [7,8]. The concept of chemoprevention has evolved to comprise interventions with specific compounds and changes in diet, in order to prevent the development of cancer (Table 1). 

Regarding cancer prevention medicine, breast and colon cancer show the strongest clinical evidence that pharmacological intervention can reduce cancer risk [13]. Recent studies reported that OPMDs are immunogenic, speculating immune-based approaches for targeted cancer prevention [14]. This work aims to analyze the possible use of drugs such as immunochemopreventive agents for OPMDs. The term leukoplakia is often used in the text because many studies in the past, including those of chemoprevention, have referred to it.

## 2. Oral Leukoplakia: Immunopathology and Rationale for the Use of Immunotherapies

Epithelial dysplasia is the most important histological finding for evaluating the possibility of oral leukoplakia progression to cancer. We analyze these lesions from another perspective and focus attention on immunopathological data. Over the past decade, there have been many advances in the immune system field. Providing a review of immunopathological aspects of OPMDs is beyond our objectives; instead, we outline some main discoveries registered since the late 1960s and early 1970s that are useful for understanding the aim of the article.

In general, the oral-pharyngeal mucosal immune system protects the oral cavity and the body against pathogens; it shares similar anatomical structure of other mucosal immune system [15,16]. 

If it is apparently intuitive to think that the oral mucosal immune system is involved in different pathological processes such as viral infection, candida infection, and systemic inflammatory and autoimmune diseases, it is perhaps less so to believe that immunosurveillance is an important factor in keeping most OPMDs in a quiescent state. Evidence suggests that the early stage of the tumor carcinogenesis process is associated with immune response changes in cytokine levels, immune cell density, and immune cell function in the context of the microenvironment [17,18]. 

Historically, the first works concerning a possible association between the role of the immune system and the neoplastic transformation of leukoplakia date back to the early 1970s. Among them, Roed-Petersen et al. discovered that oral leukoplakia was associated with hypersensitivity against antigenic components of the leukoplakia tissue [19].

Interestingly Löning et al. found a leakage of locally synthesized immunoglobulins in an altered oral mucosa and highlighted changes in the local immune system of the oral mucosa in premalignant and malignant lesions [20]. In addition, Chaudhry in 1980 reported that the overall cellular immunity was depressed in leukoplakia and, particularly, in cancer patients [21]. It was further noted that impairment of cellular immunity was more marked with advance of the disease [21]. However, a paper by Pillai et al. studied these events [22]. 

Other studies analyzed the inflammatory infiltrate in order to differentiate leukoplakia and lichen planus even if specific diagnostic criteria cannot be established [23]. 

Later, the study of the expression of Major Histocompatibility Complex (MHC) class I antigenic became an interesting field of research [24] and highlighted the role played by the cells presenting antigen (APC) initially known as Langerhans cells and currently defined as dendritic cells [25]. Dendritic cells (DCs) are antigen-presenting cells with an important role in causing a T cell response [26]. Dendritic cells promote the induction of a subset of lymphocytes T cells known as regulatory T cells (T-regs), through cytokine and noncytokine mediators [27]. 

In 1995, Bondad-Palmario described the immunocompetent cells and their distribution in oral leukoplakia with different levels of dysplasia [28]. Cells were identified in the epithelium and subepithelial connective tissue. In both compartments, there was a prevalence of T-lymphocytes. The B lymphocytes are organized as diffuse aggregates or in follicular patterns, while T lymphocytes made up the paracortical areas. A decrease in CD4/CD8 ratio in severe dysplasia cases has also been found. Mild to severe dysplasia specimens presented an increase of CD1a (+) dendritic Langerhans cells when compared with those of epithelial hyperplasia. In the sub-epithelial connective tissue of all dysplastic cases, a significant increase in macrophage count was also observed. An important increase of CD57 (+) natural killer/killer cells in the sub-epithelial connective tissue and HLA-DR expression by the keratinocytes was observed in severe dysplasia cases. 

All these results show an immune-cellular response strictly related to the degree of dysplasia in oral leukoplakia. Some immunologic phenomena, such as the reduction of CD4/CD8, the increase of HLA-DR keratinocyte expression, and the increase of natural killer cell number, are frequent in severe dysplasia and can be early biomarkers of malignant transformation [28]. 

Actually, in routine reporting, the histology of oral leukoplakia does not have hallmarks of an immunological disorder. Investigating immunological phenomena in oral leukoplakia histology, such as types and number of immunologic cells (CD8+, CD57+, etc.), could be useful to understand the possible role of immunological processes in oral transformation. 

Interestingly, some of the immunopathological features described by Bondad-Palmario could be interpreted nowadays as tertiary lymphoid structures, which are immune cell aggregates within or adjacent to areas of chronic inflammation [28]. 

In the cancer setting, the presence of tertiary lymphoid structure in the tumor microenvironment correlates with increased disease-free survival in patients [29]. Currently, several approaches are being developed to induce tertiary lymphoid structure formation [30] and this approach represents promising avenues for cancer treatment and possibly for precancerous lesions as well (Table 2). 

In addition, Gannot et al. in 2002 found a link between moderate and severe dysplasia or Oral Squamous Cell Carcinoma (OSCC), worse pathological conditions, and a higher level of immunocompetent cells, such as lymphocytes and macrophages, if compared to milder epithelial modification, such as hyperkeratosis or mild dysplasia [42]. 

Ohman et al. [43] found that the number of CD4+ cells was not different between leukoplakia with and without dysplasia, but there was an important increase in OSCC comparing to leukoplakia. CD8+ cell number significantly increased in OSCC and leukoplakia with dysplasia. There was also an increase of Ki67 positive cells in OSCC compared to leukoplakia. Langerhans cells and T cell number were higher in tissue sections with dysplasia and dramatically increased in OSCC [43]. In a successive study, Ohman et al. found that the number of CD3-expressing T cells may be important for preventing malignant transformation of leukoplakia [44]. Nevertheless, over the last years, a growing attention has been paid to the role played by another immune cells, the tumor associated macrophages, in the progression of both oral squamous cell carcinoma and precancerous lesions [45]. For example, Mori et al. identified the phenotype of tumor-associated macrophages in leukoplakia and an increase in CD163+ macrophages in oral potentially malignant disorders, showing that CD163+ macrophages co-expressed STAT1, a M1-related marker [46]. 

Interestingly Stasikowska-Kanicka et al. reported that infiltrated T cells were involved in macrophage polarization in leukoplakia and OSCC [47]. They found that the amount and localization of lymphocytes and macrophages in leukoplakia and OSCC are a possible biomarker of cell infiltration influence on the early and subsequent stage of oral carcinogenesis [47]. Additionally, Weber et al. reported an increased macrophage infiltration and M2 polarization and the association of these events with the evolution of oral leukoplakia in oral cancer [48]. 

The study of programmed death ligand-1 (PD-L1) expression and programmed death-1 (PD-1)-positive tumor-infiltrating lymphocytes became a further line of research, quickly considering the use some immunological drugs able to inhibit PD-1/PD-L1 pathway. Dave et al. studied the expression of PD-1 and PD-L1 in oral dysplasia in progression to OSCC compared to non-progressing dysplasia [49]. Their results suggest that immunomodulation using PD-L1/PD-1 pathway happened before malignant transformation [49]. Kouketsu et al., analyzing 106 OSCC and 79 oral precancerous lesions for PD-L1 and PD-1 expression by immunohistochemistry, found a significant positive correlation between PD-L1 and PD-1 as well as their expression in these lesions [50]. In particular, Yagyuu et al. highlighted that PD-L1-positive cells and epithelial PD-L1 positivity were significantly associated with malignant progression [51]. They speculated that PD-L1-positive dysplastic cells and recruited subepithelial cells in OPMDs could escape the host immune system [51]. In addition, Jiang et al. suggested TAMs (Tumor Associated Macrophages) expressed upregulation of PD-L1 and high capacity in inducing T cell apoptosis comparing to peritumoral macrophages [52]. The PD-L1 expression was directly correlated with the amount of T cell apoptosis [52].

It is interesting to observe that the migration of T lymphocytes is conditioned by the release of mediators by the precancerous cells themselves [18]. Woodford et al. showed that the microenvironment of the precancerous lesion contains inflammatory mediators and IL-17, but this inflammatory phenotype decreases as the precancerous oral lesions transform into a tumor. This effect would be associated with their reduced production of IL-23 and increased production of TGF-β [53]. Additionally, Caughron et al. showed that IL-23 can perform a pro-inflammatory function in precancerous lesions but activates an inhibitory function in the later stages of neoplastic development [54]. 

TGF-β accomplishes tumor suppression activity in normal cells and early tumors by inducing cell cycle arrest and apoptotic reactions. Conversely, in the advanced stages of tumor progression, TGF-β acts as an oncogenic factor that promotes the growth, invasion, and development of carcinoma metastases. This dual behavior in TGF-β function has been linked to the ability of TGF-β to induce epithelial–mesenchymal transition (EMT) programs. Interestingly TGF-β also plays a critical role in T cell function; this aspect represents a promising goal in cancer therapy [55]. 

A peculiar aspect concerns the possibility that smoking related lesions have a different immunological pattern. Souto et al. demonstrated that, in samples of oral leukoplakias, dendritic cell density decreases in the presence of smoking and increases in larger lesions and epithelial dysplasia [56]. However, Pentenero et al. found that a stronger relationship of smoking with lesions in the buccal mucosa and FOM than in the tongue; this observation suggests that tissue characteristics mediate the effects of tobacco [57]. Additionally, recent studies showed that, in squamous cell carcinomas, the genetic signature of smoking is associated with a higher mutational burden, but the effects on tumor immunity depend on the anatomical site. In head and neck cancers, smoking is predominantly immunosuppressive, while, in lung cancer, it is more pro-inflammatory [58,59]. 

In conclusion, the immune system plays an important role in the microenvironment of preneoplastic and neoplastic lesion; the knowledge on the role that the microenvironment plays in controlling the local aggressiveness of cancer cells as well as their growth and diffusion have progressively increased over the years and currently it could represent a target for targeted therapies [60]. However, the role played by the microenvironment in the very early stages of the malignant process seems different from the invasive stage. The data seem to suggest that the preneoplastic microenvironment is characterized by pro-inflammatory modifications [18] while the neoplastic microenvironment tends to become immunosuppressive in relation to the action of the myeloid-derived suppressor cells [61]. Furthermore, as highlighted by Beane et al. studying the persistent bronchial potentially malignant disorder, only the complete characterization of the immune populations may not be sufficient to elucidate the mechanisms of impaired immune surveillance [62]. Similarly, Krysan et al. suggested the existence of an association between adaptive immune responses and the mutational landscape in adenomatous premalignancy [63]. 

Finally, tumor mutation load and the immune microenvironment influence clinical response to immunotherapy.

## 3. Immunopathology and Field Cancerization

Field cancerization and multistep carcinogenesis represent the rationale for chemoprevention interventions [64]. Field cancerization hypothesis suggests the potential development of cancer at multiple sites; some evidence points out that, within the field, synchronous oral squamous cell carcinomas may be of independent origin in some patients, but may be of common clonal origin in others [65]. Besides genetic modifications, other theories have been proposed to explain the field effect. In their pioneering work, Ge et al. hypothesized, on the basis of published literature and their work, a new model of field cancerization centered on the co-evolution of the tumor microenvironment [66]. Currently, evidence highlights that immunological changes can affect not only the single lesion but the entire mucosa [67]. In fact, in their recent works, Zhang et al. highlighted that immune factors and cancer associated fibroblast contribute to the development of multiple primary cancers, which are considered a hallmark of mucosal predisposition to the development of cancers such as precancerous lesions [68,69]. They reported that reduction of T-cell numbers, CD3 zeta chain, and HLA class I molecules can promote the development of primary cancers by inducing immunosuppression [68]. Decreased expression of Smad3 and cJUN of tumoral fibroblasts reduces the GPX1 activity with subsequent elevation of extracellular hydrogen peroxide [68]. High hydrogen peroxide levels in the microenvironment can promote the evolution of normal fibroblast into CAF phenotype, and support multiple primary cancers [68]. Monteran et al. showed that cancer associated fibroblasts realize the immune microenvironment in tumor tissues until a pro-tumorigenic and immunosuppressive condition by affecting the recruitment and function of several innate immune cells [70]. The role of immunity in the development of malignant lesions of the oral mucosa might also emphasize the relationship between chronic trauma and the onset of malignancies. Chronic inflammation is considered a risk factor for oral cancer; in this view, Sun et al. proposed that immunosuppression due to chronic flogistic processes encourages oral tumorigenesis, rather than initiating it [71].

## 4. Mutational Load and Immunology: Should These Factors Be Analyzed in Chemoprevention Studies?

Multistep carcinogenesis can be summarized in simple way as a progressive increase of genetic damage during the transformations from the early phases of cellular atypia to frank malignancy. It is reported that chemoprevention may be not useful if the target population has a very high risk or is already affected by preneoplastic lesions with irreversible cellular changes [67]. In general, in cancer patients, there is an association between tumor mutational burden and response to immunotherapy; in addition, this relationship is cancer type dependent. Consequently, it is interesting to explore in this contest the relationship among the entities, the dynamics of genetic modifications, and the type of immune response for planning a targeted treatment. For example, the dynamics of molecular modifications induced by the 4-NQO model suggest that MEK inhibition may be important to prevent and treat a specific molecularly defined subgroup of OSCC [68]. Studies of cancer patients have clearly demonstrated a relationship between the amount of mutations and characteristics of the immune response. In general, in cancer patients, the associations between tumor mutational burden and diverse immune signature types are generally cancer type dependent. In particular, Wang et al. showed that patients with higher-TMB may have a more positive prognosis in several types of cancer types if treated with immunotherapy; even if for head and neck cancer, the lower-TMB subtype has better prognosis than the higher-TMB subtype [69]. Does the same relationship exist for precancerous lesions or does this mean that only the most advanced precancerous lesions can respond to immunological therapies even if their prognosis in general is worse? Additionally, we stress the relationship between TP53 mutation and immune signatures in head and neck cancer. Lyu et al. confronted 20 immune signatures levels of TP53-mutated vs. TP53-wildtype HNSCCs and HRAS-mutated vs. HRAS-wildtype HNSCCs [70]. They found that TP53 mutations were present with reduced immune signatures while HRAS mutations were related to increased immune signatures in HNSCC [70]. The relationship between TP53 and immune signature has been confirmed in other recent work by Li et al., indicating the connections between TP53 mutations and anticancer immunity as a consequence of the effect of the altered TMB and tumor aneuploidy level due to TP53 mutations on tumor immunity [71]. In particular, they discovered that, in HNSC, tumor aneuploidy level more strongly affected antitumor immunity than TMB [71]. It is necessary to keep in mind that early mutations in TP53, a frequent finding in leukoplakia as well as in head and neck carcinoma, are related to a copy number genomic instability [72]. On the other hand, Castagnola et al. found that only OPMDs on the tongue mucosa showed a major frequency of aneuploidy confronted to OPMDs on buccal mucosa [73], which highlights the possibility that only OPMDs limited to tongue mucosa may benefit from immunotherapy treatment. Moreover, Bhattacharya et al. found four genes associated with decreased CD8+ T-cell number in the microenvironment of tumor when transcriptionally affected by copy number alterations [74]. It is known that a large number of CD8+ T cells is associated with major response rates to immune checkpoint inhibitors [75,76]. However, Castagnola et al. also demonstrated that aneuploidy, global genomic derangement (measured as the total number of Copy Number Alterations, CNAs), and specific focal CNAs are present early in the evolution of oral cancer and are more frequent at later stages [73]. These results are in accordance with Zhang et al., who showed that, before the acquisition of invasion, immunoediting and intratumor genomic heterogeneity are early events that may happen [77]. Interestingly, Gu et al. showed that, in the tumor-free tongue of patients with SCCOT, the cytolytic activity has clinical relevance rather than the levels of immune infiltration or degree of cytolytic activity within the tumor [78]. This observation could be interesting regarding the potential efficacy of immunological drugs against mutant clones in normal appearing epithelium. The presence of several oncogenically initiated clones in normal tissues, a thorough description of which is beyond our scope, has been reported for at least two decades. However, concerning changes in gene expression profiles, a clear distinction might be made between clinically tumor-free tongues in patients with squamous cell carcinoma of the tongue and healthy controls. In fact, Boldrup et al. revealed dysregulation of 554 genes in clinically tumor-free tongues confronted to control tongues in healthy individuals [79]. On the other hand, Martincorena et al. not only showed that mutant clones can be found in normal human esophagus mucosa of subjects without cancer but, most importantly for chemoprevention, also that the quantity of mutations increase with age [80]. In particular, Blokzijl et al. showed that about 40 novel mutations per year strongly appeared in all tissue types studied, despite the many changes in cancer incidence among these tissues [81]. Risques and Kennedy highlighted that age can promote accumulation of mutation generating new clones of altered cells [82], although, in Martincorena’s experience, the number of driver mutations per cell in the healthy esophagus is much lower than that in cancer cells with a mutation burden approximately ten times lower than that in many esophagus squamous cell carcinomas [80]. Laconi et al. wrote that a big challenge will be the consolidation, with epidemiological data, of these studies, which could have a high impact on addressing cancer risk and treatment [83].

## 5. Immunology and Aging in Patient Affected by Oral Precancerous Lesion: Does a Progressive Increase in Mutational Load and Immunosenescence Concern Us?

In the prospective of immunoprevention of precancerous lesions, we also need to consider the effects of aging. As analyzed in the previous section, studies have shown that as age increases there is a progressive increase in the number of mutations in the lesions as well as in the tissues around the lesions; these phenomena regard not only passenger mutations but also driver mutations [84].

It is therefore not surprising that that a long duration of leukoplakia is an important risk factor for the malignant transformation. It should also be remembered that the immune system seems to perform more poorly in older patients and could negatively influence an immune-centered treatment. This aspect relates to the fact that age can influence immune surveillance, which is known as immunosenescence. Immunosenescence occurs in healthy subject and in cancer patients too. Jeske et al. suggested that, in elderly HNSCC patients, the immune system is damaged and the tumor-induced immune escape is less present [84]. Jeske et al. showed an alteration of immune competence due to age, such as lower number of CD8+ T cells and reduced CCR7 and higher PD1 expression [84]. However, the increased expression of PD-1 is an important factor for effective immunotherapies in the elderly; for this reason, checkpoint inhibitors could be more useful in older patients with head and neck cancer [84]. Similar studies are not available for elderly patients with preneoplastic lesions, while clinical studies show that duration of leukoplakia [85], age of patients, and size of lesions are important prognostic factors for oral carcinogenesis [86].

## 6. Chemoprevention of Oral Leukoplakia: Basic Concepts

Experience coming from the pioneering chemoprevention studies with 13-cis retinoic acid can help us to highlight the boundary of this topic. Sporn definition of chemoprevention included the use of drugs to reduce/eliminate preinvasive intraepithelial precursor lesions which evolve to invasive cancer [7]. Although the elimination of intraepithelial neoplasia with drugs could be considered as the “therapy” of neoplasia, as shown by Sporn definition, it is customarily viewed as the prevention of invasive neoplasia, or cancer [7]. Much promise has been shown in this field. However, a great evolution in the philosophy of chemoprevention of oral cancer was observed in the early conclusion of Richtsmeier [87] We can now treat what was formerly called Commended mucosa as if it had been given at least a temporary reprieve. following the initial observation of the effect of 13-cis retinoic acid to the subsequent observation reported by Papadimitrakopoulou et al. that Our results did not establish short-term oral precancerous lesions response as a surrogate end point for oral cancer free survival [88]. This discovery shows the potency of potentially malignant disorder response as a surrogate for cancer development [88]. In fact, the early clinical response of oral leukoplakia treated with retinoic acid reported by Hong et al. in their original report did not correspond to a change in biological behavior during follow-up [89]. This phenomenon was summarized by Mao et al., who showed a discordance between the clinical and histologic responses and the genetic status, proving a lag between the phenotypic and the genetic responses to the treatment [90]. The lack of chemoprevention activity due to isotretinoin was further [91] demonstrated by Khuri et al., displaying that isotretinoin was not efficacious in mediating chemoprevention in patients with HNSCC early-stage [92]. However, more recently, similar results were also found by Bhatia et al., who reported that a treatment with low-dose 13-cis RA for two years did not reduce the incidence of Second Primary Tumors [93]. Based on the initial experience with 13-cis retinoic acid, Papadimitrakopoulou et al. proposed a combinational chemoprevention trial, defined as biochemoprevention, based on the combined use of 13-cis retinoic acid, alpha interferon, and alpha tocopherol as a promising biologic approach for laryngeal dysplasia [9]. Moreover, lesions of the oral cavity presented 9% of remissions after six months [9]. The search for agents with complementary mechanisms for suppressing the process of carcinogenesis is currently an exciting line of research [37,94]. Interestingly, among agents tested on the combination use, there are also drugs with immune activities. Another milestone study in chemoprevention of oral cancer useful to clarify the evolution in planning clinical studies was a recent clinical trial on the use of erlotinib in oral cancer prevention [95]. This trial represented the first protocol of precision medicine in cancer prevention [95]. In fact, loss of heterozygosis is an important marker of oral cancer risk and linked to increased EGFR copy number [95]. However, erlotinib did not reduce the risk of oral cancer in patients with LOH-positive oral precancerous lesions [95]. The timing and dose of drugs can influence response. Naturally, the action of these drugs is be not useful in high risk population or those with preneoplastic lesions characterized by irreversible cellular alterations [67].

## 7. Immunoprevention: Possible Role of Vaccination in Preventing Preneoplastic Oral Lesions

From a general point of view, human cancer immunoprevention can be divided into two types: tumors related to infectious agents and tumors unrelated to infectious agents [96]. Nonetheless, to simplify our description, we can also divide immunoprevention through vaccine and immunoprevention with drugs. Regarding head and neck tumors related to infectious agents and the employment of vaccine, the most common vaccination analyzed is against high risk-HPV. Briefly, in head and neck cancer, oropharynx is the most common localization of HPV related tumors. For other sites, the oncogenic role of HPV is less clear and the use of vaccination to prevent malignant transformation may be questionable. These types of vaccines target the protein L1 in order to prevent infection [31]. The expression of L1 antigen is lost once HPV completes its integration into genome of the host and this event allows these vaccines improbably to create a protective response against established HPV+ cancer [32]. In addition to the prophylactic vaccination against human papillomavirus (HPV), therapeutic vaccines against HPV with one or more immunomodulatory agents are being tested [97].

However, vaccination was also studied for immunoprevention of oral precancerous lesions. In particular, Young explored the possibility of using cell-based vaccines using/utilizing pulsed dendritic cells in potentially malignant disorder; she found that vaccination may be effective in the reduction of potentially malignant disorder cell burden and in the increase of protection from an OSCC [33]. Nonetheless, immune evasion could reduce the efficacy of dendritic cell vaccines. De Costa et al. conducted a study to establish whether an immune response can be aroused by administering a dendritic cell vaccine during the premalignant stages prior to development of immune escape [34]. They found that dendritic cell vaccination may have a positive effect on clinical outcome, regardless of type of antigenic stimulation [34]. One possibility of increasing the efficacy of vaccines is represented by the use of adjuvants such as cyclic dinucleotides that act as Stimulation of Interferon Genes (STING) agonists [98]. Furthermore, Baird et al. showed that STING could be found in the basal layer of normal tonsil and in tonsillar crypts [36]. Additionally, cyclic dinucleotides could be used for immuno-prevention of cancer and precancer HPV + [36].

## 8. Immunotherapy: Can the Modulation of the Immune Response through the Use of Drugs Help in the Prevention of Preneoplastic Lesions?

Going beyond the specific role of cluster cells or cytokines [46], the data of this brief analysis describe the existence of a relationship between the activity of the immune system, sometimes also of an inhibitory type, and the evolution of precancerous lesions of the oral cavity. This might represent the rational basis for the use of immunomodulators such as chemopreventive agents. The principal aim of immunotherapy is to act on natural immunosurveillance and induce the tumor destruction or at least the equilibrium with immune system.

There are several lines of evidence that many cancer chemoprevention agents, such as aspirin, COX-2 inhibitors, aromatase inhibitors, and bisphosphonates, act by enhancing immunosurveillance and/or inverting the immune evasive processes that potentially malignant disorders induce.

Johnson and Young evaluated the efficacy of indomethacin using the 4-NQO mouse model of oral carcinogenesis. This study indicates that inhibition of prostaglandin production in the precancerous lesion phase increases immune capacity and improves clinical outcomes [99].

The use of PD-1 inhibitors appears interesting in patients with advanced disease of the head and neck area; several experimental studies also seem to suggest the possible use of PD-1 inhibitors as drugs for prevention. To explore this possibility, Levingston et al. used a model of a carcinogen-induced precancerous oral lesion [100]. This experimental study, while demonstrating an early response to treatment with PD-1 antibodies, did not demonstrate the possibility of preventing over time the possibility of preventing neoplastic progression [100].

As regards the clinical trials, a randomized phase II trial study of immunotherapy with pembrolizumab in high risk oral intraepithelial neoplasia is underway [38]. The primary objective of this study was to determine oral cancer-free survival of high-risk patients with oral intra-epithelial neoplasias (IEN) treated with pembrolizumab versus observation [38]. Secondary objectives were the evaluation of safety and tolerability of pembrolizumab for patients with oral IEN, the histologic and clinical response rates to pembrolizumab, and finally to characterize the immune infiltrate in oral IEN lesions before and after treatment with pembrolizumab [38]. Four administrations of Pembrolizumab are given to patients affected by oral IEN and characterized by molecular high-risk profile of LOH, such as 3p14 and/or 9p21 and other chromosomic alterations [38]. However, the route of administration, the elevated cost of PD-1 antibodies, and the high risk of toxicities might limit the clinical efficacy of this treatment [38]. Emerging evidence shows that inhibition of mTOR could influence the anti-tumor immune response. Even though the PI3K/mTOR pathway is the most frequently started up, these new results show that the combination of immune oncology agents and mTOR inhibitors could provide novel precision therapeutic options for HNSCC [101]. An attractive scenario is given by the possible topical administration of mTOR. An article reinforces this hypothesis; in fact, Nudelman Z. et al. analyzed the levels of sirolimus in saliva and plasma in patients who take this drug chronically in order to evaluate total oral exposure [102]. They found saliva drug levels on average six times lower than those in plasma and concluded that this observation represents the rationale for topical administration of sirolimus in patients with oral cancer [102]. Several studies are listed in the literature concerning the topical use of tacrolimus, a drug with immunosuppressive activity and structurally related to sirolimus, in the symptomatic therapy of lichen planus of the oral mucous membranes, a disease which presents analogies with types of alterations that are precancerous. In general, the results of the experiments conducted were encouraging but we want to emphasize that the topical use on oral precancerous lesions has not yet been approved. Rautava J and others studied the effect of tacrolimus in an in vitro model that simulates the oral mucosa (raft culture model) [39]. They found that, although the in vivo pharmacological treatment is accompanied by a symptomatic response that occurs within 24 h of treatment, it does not cause modifications in several factors involved in oral carcinogenesis [39]. In a systematic literature review, Elad S et al. highlighted the lack of evidence that supports the use of sirolimus in lichen planus, while favorably evaluating the few findings found in the literature [103]. An interesting study concerns the topical application of sirolimus in three cases of pemphigus vulgaris of the oral cavity [40]. The authors treated selected patients topically with sirolimus using 5 mL of a 1-mg/mL solution of sirolimus twice a day as a rinse [40]. In one patient, sirolimus was used with a reduced dose of prednisone 20 mg/day, while in the other two no other drugs were used [40]. The patient plasma levels of sirolimus evaluated after eight days were not evaluable [40]. The rinsing was well tolerated in all patients without local irritation and the treatment lasted for 2–3 weeks [40]. However, no symptomatic improvement was noted in any patient during treatment [40]. Another interesting study concerns the topical application of sirolimus in pediatric patients with tuberous sclerosis [104]. Four patients with sclerosis tuberosis were recruited to receive topical 0.1% rapamycin [104]. Two patients were treated with 0.1% rapamycin in petrolatum (vaseline) to be applied twice a day and two patients with a 0.1% solution [104]. A 0.05% Desonide solution was used to prevent local irritations [104]. Blood samples were performed at one, two, and six months to evaluate systemic absorption [104]. A significant response was recorded in all patients as early as the first week of treatment [104]. The younger patients exhibited the most significant response [104]. The rapid initial improvement continued over the successive six months [104]. An interesting study concerns the topical application of sirolimus in seven female patients with erosive lichen planus [105]. Soria A et al. studied the topical application of sirolimus in seven patients with erosive lichen by administering a solution of rapamycin (1 mg/mL) on the lesions twice a day for three months [105]. Four patients also had vulvar lesions and the same solution was applied to these lesions [105]. Rapamycin plasma levels were also analyzed after fifteen days. At three months, four patients had complete remission and two had partial regression. One patient stopped treatment due to local discomfort [105]. Rapamycin plasma levels were recorded in only one patient [105].

## 9. Discussion and Conclusions

Oral leukoplakia is considered a good model for chemoprevention intervention and, since the 1980s, many clinical studies have been conducted with different agents to treat leukoplakia in order to prevent the evolution of oral cancer. High degree of intraepithelial neoplasia was one of the biomarkers, even if not the only one, and the most used to test (to validate, to prove) efficacy in the protocol of early treatments. However, the vast increase in knowledge resulting from cancer research in recent decades made it possible to identify with greater accuracy the varying degrees of disparate genetic, molecular, transcriptomic, and immunologic profiles that characterize preneoplastic lesions. Some therapeutic strategies for the molecular cancer prevention are the use of natural or synthetic compounds that stop the prime drivers, key imbalances, or the context in which these drivers act and in which the derangements occur, prior to invasion across the basement membrane [11,77,106,107]. Among therapies targeting head and neck cancer, immunotherapy has gained attention in recent years, not only for treatments of advanced diseases but also as molecular prevention therapy [11,77,106,107]. However, except for the HPV vaccination for oro-pharyngeal cancers, despite their conceptual promise, the efficacy of immunoprevention of oral cancer is not currently demonstrated.

Nonetheless, it is possible to point out some topics. Immunoediting is a common early phenomenon during the development of a precancerous lesion but, as highlighted by Foy et al. [108], patients may benefit from specific personalized prevention interventions in relation to subtypes of oral potentially malignant disorders [108]. In general, chemoprevention requires long-term efficacy; the timing, dose, and toxicity of agents should be closely considered. For example, Formelli et al. [109] showed that Fenretinide plasma concentrations were four times higher than those reached with the administration of high but intermittent doses [110]. Even in the case of proven efficacy of chemoprevention treatments, some aspects such as the improvement in the risk–benefit balance, the individuation of minimal active doses, the possibility of particular treatment strategies, a precise individuation of the risk categories, and the containment of costs remain fundamental [111]. From this prospective, the use of PD-1 antibodies as chemopreventive agents for high risk oral lesions, due to i.v. administration, risk of high-grade toxicities, and high cost of PD-1 antibodies, could be limited [112]. Additionally, patients receiving PD-1 inhibitors may develop oral immune-related adverse events characterized by lichenoid lesions, ulcers, or erythema multiforme [113]. Moreover, other classes of targeted anti-cancer therapies with immunologic activities as mTOR inhibitors are known to induce stomatitis. In a review, Lo Muzio et al. showed that the incidence rate of stomatitis based on the agent used was 54.76% for ridaforolimus, 27.02% for temsirolimus, and 25.07% for everolimus even if the onset of severe stomatitis (G3-G4) was uncommon [114]. Concerning a way to improve the adherence (acceptability) of patients to the chemopreventive protocol treatment, a recent study by Samimi et al. indicated that patients could be more receptive to locally delivered chemopreventive compounds if they believe these substances useful for the treatment of cancer [115].

Two treatment options can be considered to reduce the systemic toxicity and improve the local amount of drug or target drug. As suggested by Nieto et al., a first option is to use alternative methods to deliver chemopreventive drugs to the oral mucosa [116,117]. The second option is the combined use of an agent endowed with complementary antitumor activities. This approach was indicated by Mallery et al. as multifaceted chemoprevention [37]; in this context, it is noteworthy that, among the agents selected, reparixin was chosen for its abilities to interfere with IL-8 signaling [118].

However, various evidence suggests that the enhancement of natural immunity or vaccine-induced immunity against cancer or infections could be achieved through the modulation of retinoic acid signaling related to vitamin A supplementation in diet, treatment with agonists, or inhibitors of retinoic acid receptors [41]. Furthermore, the research conducted by Osei-Sarfo and Gudas shows novel evidence of retinoic acid and bexarotene on the expression of FOXO3A and FOXM1 about transcriptional regulatory routes of human OSCC [119]. Alternative ways to deliver drugs, new dosage schedules, combined use of molecules with complementary antitumor activities, diet influence, and better definition of individual risk factors must also be considered to reduce toxicity and improve compliance (adherence) to the protocol [120]. In addition, Jie et al. pointed out that All-trans retinoic acid (ATRA) can determine anti-cancer actions and reduce PD-L1 expression through inhibition of STAT3 signaling in both OSCC and oral dysplasia [121]. However, these recent observations on the role of retinoic acid on the immune system cannot be completely separated from an observation underlying many chemoprevention interventions: the protective role of diet in head and neck cancer. Wide debate still exists today regarding the superior role of isolated components of diet versus diet as a whole [122]. However, Eastham et al. reported that phenolics (such as resveratrol, EGCG, curcumin, quercetin, and honokiol) and glucosinolates (e.g., sulforaphane, PEITC, and BITC), are resulting as potent and efficacious inhibitors of oral carcinogenesis. They also emphasize the concept of green chemoprevention: this is a recent approach to point out healthy lifestyle changes that aim to reduce the HNSCC incidence [10]. Green chemoprevention expects the use of natural substances in order to avoid the epithelial malignant transformation. Several clinical trials on tea, lycopene, and beta-carotene were performed for oral cancer chemoprevention. Systemic treatment with green tea extract showed no evidence of benefit in terms of clinical resolution of leukoplakia when compared with the control in one study at unclear risk of bias (RR 0.99, 95% CI 0.8–1.14; 39 participants) [123]. Li et al. investigated a treatment with extract of green tea (green tea polyphenols and tea pigments) and topical preparations of mixed tea, but was not able to demonstrate benefit when compared with placebo in terms of clinical resolution (RR 1.00, 95% CI 0.94–1.07; 59 participants) [124]. Other studies investigated the possible use of lycopene in the oral leukoplakia treatment and pointed out that oral lycopene appears to be effective in the treatment and management of oral leukoplakia [125,126].

The comparative evaluation between the effectiveness of the diet and the use of vitamins at pharmacological doses is beyond our scope; it would be sufficient to remember that, even today, on the basis of the results of the Alpha-Tocopherol Beta-Carotene (ATBC) Study, pharmacological supplementation with the natural micronutrient beta-carotene would be not recommended for heavy smokers [127]. On the other hand, if one wishes to take pharmacological dosages of the diet component resveratrol in the diet, between 500 and 2000 L of red wine should be taken per day [128].

In conclusion, experimental and in vivo data offer us the promise of molecular prevention through immunomodulation; however, currently, there is no clear evidence for the efficacy of this drug in the chemoprevention action. Alternative ways to deliver drugs, combined use of molecules with complementary antitumor activities, diet influence and better definition of individual risk factors must also be considered to reduce toxicity, improve compliance (adherence) to the protocol treatment, and offer a better individualized prevention. In addition, we have to carefully reconsider the mode of action of many traditional cancer chemoprevention compounds actives on the immune system, in order to increase immunosurveillance and reverse the immune evasion [129].

## Figures and Tables

**Table 1:** Definitions of chemoprevention over time. Currently, there is no clinical evidence that pharmacological intervention can reduce cancer risk of malignant transformation of oral premalignant lesions. Taking in account the definitions, it is interesting to note that there has been a progressive translation from interventions based mainly on the theory of multiphasic carcinogenesis and with predominantly epithelial involvement to forms of treatment more aimed at the microenvironment as a whole. In this view, the hypothesis of modulation of immune response for prevention can be considered part of this process.

Definition	Principal Target	Date	Ref.
Chemoprevention is the use of synthetic or natural compounds for the reversal, suppression, or prevention of a premalignant lesion conversion to an invasive form.	Therapy of intraepithelial neoplasia	1976	[7]
Biochemoprevention	Combined use of interferon, 13-cis retinoic acid and alpha tocopherol	1999	[9]
Short-term intermittent therapy to eliminate premalignancy (SITEP).	Intermittent dosing schedules: (A) toxicity reduction (B) periodic reduction of premalignant cells	2011	[10]
Molecular prevention: the use of natural or synthetic agents that interrupt the prime drivers, key derangements or the context in which these drivers act and in which the derangements occur, prior to invasion across the basement membrane	Genetic, epigenetic.	2015	[11]
Green chemoprevention as a modern approach to highlight healthy lifestyle changes that aim to decrease the incidence of head and neck cancer.	Typically, is believed to act through epigenetic influence.	2018	[8,12]

**Table 2:** Report of some studies about immune chemoprevention of oral leukoplakia.

Type of Drug	Route of Administration In Vivo	Type of Study	Target in the Study	Major Results	Ref.
HPV vaccine	Parental	Clinical	High risk HPV	Efficacy not proven for oral preneoplastic lesion prevention	[31,32]
Dendritic cell vaccine	Parental	Experimental	Potentially malignant disorder-pulsed dendritic cell	Induction of immune reactivity	[33]
Dendritic cell vaccine	Parental	Experimental	(A) Potentially malignant disorder-pulsed dendritic cell vaccine; (B) Normal tongue epithelium lysate-pulsed dendritic cells vaccine	Reduction of lesion burden at 8 weeks; Rapid increase (B) or delayed increase (A) in stimulatory immune effectors	[34]
Cyclic dinucleotides	Parental	Experimental	Stimulation of interferon genes (STING)	Vaccine adjuvant; promoting both T cell and humoral responses.	[35]
Cyclic dinucleotides	Parental	Experimental	Stimulation of interferon genes (STING); interestingly STING could be found in the basal layer of normal tonsil and in tonsillar crypts.	Regression of papilloma. Immuno-prevention of cancer and precancer HPV+	[36]
Alfa interferon	Parental	Combinational chemoprevention trial. Combined use with 13-cis retinoic acid and alpha tocopherol	Stimulates the production of enzymes modulating the immune response	Moderate effectiveness for laryngeal dysplasia. Poor response oral lesions	[9]
(A) Tocilizumab (B) Reparixin	(A) Parental (B) O.S.	Experimental, combination treatment with Fenretinide, tocilizumab and Reparixin in vitro.	(A) Humanized monoclonal antibody against the interleukin-6 receptor. (B) Inhibitor of the chemokine receptors CXCR1 and CXCR2	Treatment significantly suppressed IL-6 and IL-8 release, stem cell gene expression, and invasion	[37]
Pembrolizumab	Parental	Randomized phase II trial study. Start Date: 14 June 2017 Estimated Primary Completion Date: 1 March 2024	Humanized antibody. Programmed cell death protein 1 (PD-1) receptor.	Results not available	[38]
Tacrolimus	Topical	Experimental	Calcineurin inhibitors with blocking the production and release of pro-inflammatory cytokines in T cells, and improving the barrier function of the skin and the mucosa	No reduction of factors related to malignant transformation unchanged	[39]
Sirolimus	Topical	Clinical	m-TOR inhibitor 5 mL of a 1 mg/mL solution of sirolimus twice a day as a rinse	local anti-inflammatory effects	[40]
Dietary supplementation with vitamin A, treatment with agonists, or inhibitors of retinoic acid receptors	Systemic	Experimental-Clinical	Natural and vaccine-induced immunity against infections or cancer. Modulation of retinoic acid signaling	Modulation of immune activity	[41]
